# Creating small-area deprivation indices: a guide for stages and options

**DOI:** 10.1136/jech-2019-213255

**Published:** 2019-10-19

**Authors:** Mirjam Allik, Alastair Leyland, Maria Yury Travassos Ichihara, Ruth Dundas

**Affiliations:** 1 MRC/CSO Social and Public Health Sciences Unit, University of Glasgow, Glasgow, UK; 2 Center for Data Integration and Health Knowledge, Salvador, Brazil

**Keywords:** deprivation, measurement, health inequalities, social epidemiology, socio-economic

## Introduction

The creation and use of composite indices for capturing various complex or multidimensional concepts (eg, deprivation, geographic access, green space, sustainability, corruption, transparency) has become extremely popular. Measures are created at national, regional or small-area level. There is considerable literature on creating measures at the national level, much of it critical, discussing the different implicit (and potentially biassed) decisions taken by researchers or organisations creating the measures.^1^ Few researchers discuss the different choices available or the steps taken to create small-area measures and the subsequent effect of these decisions on the results.^2–5^ More often, measures are created with little discussion or justification of the methods, let alone validation or uncertainty.

We believe there is scope to review and evaluate the steps and options available when creating composite measures. As an example of the process of developing a small-area measure, we are focusing on deprivation measures in public health research. Small-area deprivation measures have been widely used to understand inequalities in health in the UK since the 1980s^6–8^ and have since become common in many other countries.^9–12^ They are appealing as they help summarise complex phenomena into a single numeric representation, are easy to use and allow robust national level analysis with aggregate data. Nevertheless, the seemingly simple composite measures belie many important decisions explicitly (or sometimes implicitly) taken by the researchers. Our aim is to outline the key stages and options available to researchers, and to discuss their potential merits and problems.

## Developing deprivation measures: stages and options

Historically, small-area deprivation measures have aimed to locate areas (and the people living in these) on a scale of material well-being,^6 13^ but more recently this has also extended to the physical environment.^14^ The measures generally cover multiple different dimensions of deprivation, often called domains. Common domains include employment, income, social class or socioeconomic status, education and housing. Some measures include a single indicator or variable for each domain (eg, per cent of people with no high school diploma is used as the education domain),^15^ while others combine multiple indicators from the same domain into a domain scores (eg, average grades, attendance and entry into higher education are combined to form the education domain),^13^ which are then combined into a deprivation measure.

**Figure 1:**
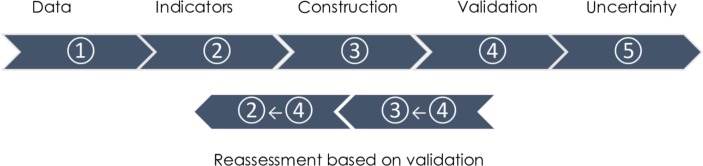
Key stages for developing small-area composite measures.

The framework for creating a deprivation measure can roughly be split into five key stages, outlined in figure 1:

1. Selection of appropriate data and geographic area.
2. Selection of individual deprivation indicators.
3. Constructing the index: combining and weighting indicators.
4. Validation and sensitivity analysis.
5. Dealing with uncertainty.

For each stage, researchers need to make decisions about the options for analysis, such as adopting a particular method. In some cases, we can make a priori decisions as to the optimal methodology or approach; in others, there is a need to examine the options empirically and an iterative approach may be necessary before a decision can be taken. Through-out the process researchers should justify the decisions made so others can understand the strengths and limitations of their approach.

### Selection of appropriate data and geographic area

Deprivation measures generally use three types of data: census,^6^ administrative^16^ and/or geospatial data.^14^ The selection of data is limited significantly by availability in terms of population coverage and completeness, and the different sources all have their own benefits and problems. The census is one of the most common sources given its accessibility and population-wide coverage, but it can be limited in terms of the indicators that directly measure material deprivation. Administrative data collected routinely by government departments are more varied in the number of indicators and may be updated more frequently.^3^ But to avoid issues relating to policy differences or change, longitudinal and cross-national research tends to rely on census data.^17 18^

Both administrative and census data may be affected by statistical disclosure control or data sharing policies, meaning that deprivation cannot be measured for some areas.^12^ For these reasons, geospatial data are appealing, especially in high-income countries where these data are more readily available.^19^ However, these can be expensive to purchase or multiple data providers may need to be approached.^10^ Sometimes data coverage is inconsistent, for example, available only for private but not public services,^20^ and there is mixed evidence on whether the accuracy of geospatial data is related to deprivation itself.^19^

All data sources have limitations and potential biases that should be considered and clearly stated. The best data source for any country will be one that most closely meets the criteria of including indicators that reflect an aspect of deprivation experienced by not just a small number of people, being up-to-date and renewable, statistically robust and collected for the whole of the country in a consistent form.^3^

In choosing a suitable geographic area, those with the smallest possible population size are preferred as this is likely to mean better homogeneity among the population and reduce the risk of ecological fallacy. There is consistent evidence that health inequalities are measured as larger when smaller areas (in terms of population) are used compared with larger ones.^11^ In reality, the choice is limited by data availability and researchers may be forced to use larger and potentially more diverse areas. In such cases, assessment of heterogeneity and its potential effects on the outcome should be given during the validation process.

### Selection of individual deprivation indicators

Selection of individual variables for any measure should be based on the theoretical fit with the concept we are interested in measuring, such as material deprivation, and on the particular context of a country. Most deprivation measures include domains relating to income, employment, socioeconomic status or class (often based on job type), education, housing and ownership of specific goods or items. Some measures also include domains relating to access to various services (schools, shops, doctors) or information on the environment (street lighting, crime rates).

There are strong theoretical grounds to include each of the listed domains in deprivation measures. For example, low-income and unemployment reflect deprivation as they limit material resources, while low levels of education disadvantage people in accessing many resources, such as better jobs or services. Sound theoretical mechanisms also connect these domains to health, for example, income curbs access to factors (eg, food, housing, services) that directly influence health and unemployment can impact health through lack of resources, social isolation, stress and loss of self-esteem. Comparisons of indicators and measures have shown that those with a strong foundation in theory are better able to explain variations in health.^15 21^

The domains and the individual variables should also be selected to capture different (though often related) aspects of deprivation and their theoretical relevance may vary by health outcomes and stages in the life course.^21^ As a result, the combined measure should be better able to capture the unmeasured concept of deprivation than the individual indicators themselves.^5^ Empirically, this should be reflected in the composite measure having a stronger association to the outcome of interest than any of the variables on their own.

While the domains included in measures are often quite similar, the actual indicators vary widely across countries. Education has been measured as literacy rate,^22^ heads of households with less than a year of education^23^ or entry to higher education.^13^ Alongside theory, country specific knowledge and the spatiotemporal context should guide the selection of indicators as deprivation is relative to what is customary to the societies in which people live.^24^ Researchers have imported deprivation indicators from other countries, but these do not always reflect the concept of deprivation in the country at hand.^9 12^

Some deprivation measures only include one single indicator per domain, but others include multiple indicators for each domain and then combine these indicators into a domain score.^10 13 16^ Multiple indicators tend to be used by those who have access to administrative data sources but are often not possible for indices based on the census, as these generally only ask one or two questions relating to each domain. The benefit of multiple indicators per domain may be the wider range of disadvantageous circumstances covered. Researchers should however be aware of and avoid double counting, that is, using identical indicators more than once in the same measure.^25^ The inclusion of any additional indicators should be underpinned by clear theoretical reasons,^3^ and adding more variables should not be an aim in itself, especially as there is no evidence that having more indicators per domain improves the measurement of deprivation.

Empirical considerations can also be helpful in selecting precise variables and choosing a definition that allows sufficient variation between areas and is neither too rare nor too common. There are no defined cut-offs, but in the 1980s and 1990s 40%–50% of people lived in social housing in Scotland^26^ and at the time the variable was excluded from the Carstairs deprivation measure as it was too common and varied little across areas.^6^ By 2011 the proportion of people in social housing had fallen to about 20% and has subsequently been included in deprivation measures.^15^

A few deprivation measures also include variables, such as ethnic breakdown, number of young or elderly people, single parent households or even disability.^27–29^ We do not recommend including such variables in deprivation measures as it is important to distinguish between deprivation and the people experiencing this.^24^ While minority populations or single parent households may experience material deprivation, being in any of these categories does not necessarily make them deprived. This is not to say that ethnicity, gender, other similar categories and their relationship to material deprivation have no relevance to health or inequalities in health. The intersection of multiple disadvantages can simultaneously shape health and health behaviour,^30^ and given the diverse nature of societies today, population health researchers need to consider the interrelationship between the different dimensions of disadvantage.^31 32^ However, these categories need to be kept distinct from material deprivation because the theoretical links that connect each of these to health may be different from that of deprivation. This can also lead to very different empirical associations, as illustrated by research in Canada, which found the impact of ethnic concentration on health to be completely opposite to that of material deprivation.^32^

### Constructing the index: combining and weighting indicators

Data availability often limits the choices researchers can make in steps 1 and 2 outlined above, but more options are available when it comes to combining the indicators into a single measure and giving the domains or the indicators weights.

#### Combining standardised scores of variables

The purpose of calculating standardised scores, such as z-scores, is to put the variables on the same scale by giving them similar means and SD. It is one of the earliest methods of constructing deprivation measures, used for the Carstairs, Townsend and Jarman indices.^6–8^ It is still used as it is straightforward and easy to replicate across time, space and geographic scale.^9^

Different weights can be applied to the standardised scores; the Carstairs score weights all indicators equally, but the weights in the Jarman index vary from 2.5 to 6.62. Equal weights have been justified with the lack of pre-existing knowledge on the importance of the indicators on the unmeasured concept of interest.^20^ Expert opinion,^29^ policy focus^7^ and individual level empirical evidence on socially perceived needs^18 33^ have also been used as a basis for weights. All of the above approaches to weighting have been classified as normative,^34^ depending on value judgements.

#### Principal component and factor analysis

A number of statistical methods can also be used to combine variables into a single or sometimes two unobserved summary measures. Different variants of factor analysis and regression-based methods can be used,^35 36^ but the fundamental logic behind these is the same—the weights assigned are data driven as opposed to normative.^34^ These methods are very popular. It is argued that they are objective; they do not require the researcher to make a judgement about what variables should be included or what influence they should have on the final measure.^12^ However, there is also no clear theoretical basis to the weights assigned to the indicators. Choosing a factor that best explains the variation in the data does not mean that the theoretical concept is explained to the same degree.

The weight of single indicators on the overall factor can vary greatly. Researchers sometimes remove the indicators with a weaker impact from the measure to construct a more parsimonious index.^27 36^ In other cases, all indicators are left in, but their effect on the summary measure will vary. Bonfim *e* *t al* 23 use seven indicators to create a deprivation measure, five of which relate to housing conditions and then education and income. The first five have factor loadings between 0.75 and 0.89, education 0.24 and income 0.47. It is difficult to argue that education is included in the measure in a substantive manner; rather the multiple deprivation measure is reduced to a housing deprivation index. In such instances, researchers should explicitly state that some domains or variables will have a smaller and others a more substantial impact on the measure and include justification for their decision.

A major shortcoming of factor analysis is poor replicability across time and space. Correlations between indicators vary across time, space and geographic scale, meaning that the different indicators will have different weights at different time points and for different levels of area aggregation. This makes factor analysis less suited for longitudinal research, or for work that aims to develop a deprivation measure for different countries or levels of analysis. For example, Pornet *et al* 37 develop a European Deprivation Index for France with the view to extend it to most other European nations. They use logistic regression to create weights for the 10 components of the French index. The same regression on the data from another European country would likely produce different weights. Would all the European countries then use different weights for the same indicators and is that desirable for a single European wide index? While it may be possible that different weights (and even indicators) should be used for different countries to reflect their specific circumstances,^1^ this knowledge is unlikely to come from statistical analysis alone, and should rather be guided by theory and the specific context of each country.

#### Developing domains from variables and combining domains

This is a strategy used for indices of multiple deprivation that combine a large number of single indicators from multiple data sources.^10 13^ Indicators that relate to the same domain, such as information on policy take-up rates that all relate to employment, are combined into a domain score and the domains are then combined into a single measure. Since different weights and methods of combining indicators into domains can and sometimes should be applied, the number and complexity of decisions to create these types of indices is considerable.^3^

There is no single widely accepted framework for choosing weights or combining indicators into indices or domains, and it will be up to the researchers developing and using deprivation measures to consider the pros and cons of the different approaches. It is likely that the final decision on weights can only be made after validation and sensitivity analysis.

### Validation and sensitivity analysis

Any composite measure should be validated to check the measure captures what was intended and does so equally well for different subpopulations. This process also allows researchers to revisit some decisions made earlier about the choice of indicators, weights and/or methods of constructing the measure. Unfortunately, there are not many methods available for validating deprivation measures, which is part of the reason why so few researchers who develop small-area measures explicitly discuss validation.^4^ The methods most easily available to validate indices include correlation between the indicators, to other similar measures and to outcomes that the measure is either intended to predict or might associate with.

Correlation of the developed measure to other known similar indices and correlation of the component indicators to each other and to the overall measure should be strong, though unlikely to be perfect. A problem with this approach is that there is no gold standard against which to test the measure or the indicators and no defined empirical cut-off as to what is a strong enough correlation. Regardless, correlation, principal component or factor analysis can all be effective in eliminating variables from the measure.^36 38^

Testing deprivation indicators and the final measures against health outcomes might be one of the best approaches for validation. This is most useful when the relationships between the different indicators and the health outcomes are compared.^39 40^ It could be argued that indicators that are best at distinguishing between the different levels of deprivation are those that are also best at describing the variation in health. Thus, a measure performs well in capturing deprivation if it can explain differences in health.

If the developed measure has a weak correlation to outcomes of interest, but appears sound otherwise (eg, in terms of theory, data and methodology), researchers should consider the population size and potential heterogeneity of the small areas. The measured deprivation of an area is always an average across its residents, and as the population size and heterogeneity of the area increases, this average is less likely to reflect the actual material well-being of the people. It might be useful to look at the variation in the individual deprivation indicators, for example, unemployment, across the small areas. If this appears low and contrary to common country specific knowledge, then the areas might be too heterogeneous to accurately capture the full scale of deprivation. In such cases researchers should note that the results, such as socioeconomic inequalities in health, may be underestimated.

Robust measures should also have explanatory power across different contexts, such as being able to identify deprivation in urban and rural areas, ethnically diverse and homogenous areas, and detect differences in health across age groups, gender and so forth. Greater application of intersectional approaches can improve validity,^31^ but analysis of this kind for deprivation measures is rare. Researchers who have compared the performance of deprivation measures or indicators across population groups have found significant differences between them in predicting health inequalities for some population groups.^15 36 41^ Because of this, future work on small-area indices should pay more attention to testing measures across different contexts.

This stage also provides an opportunity to re-evaluate the selection and combining of the indicators into a single measure (steps 2 and 3 above). If the performance of an indicator varies significantly by population groups, it could be a sign that for some people or areas the variable does not capture deprivation. There may be good theoretical reasons for this, for example, car ownership in rural areas is often argued to be a necessity rather than a reflection of material well-being.^42^ When contextual differences between areas are significant, different definitions or coding decisions (such as for urban and rural areas) can be used for the same domain.^43^

### Dealing with uncertainty

All deprivation measures are an estimate of ‘true’ deprivation that cannot be measured directly and because of this, some method should be applied to either minimise or measure uncertainty. This is particularly the case for small areas, where researchers are often dealing with very small numbers of events.

Confidence intervals provide a measure of uncertainty and can be derived using different simulation methods. Two different methods have been applied for the most recent Carstairs scores: (1) the weights attached to each indicator were varied to account for uncertainty in the influence each indicator has and (2) the counts of events for all indicators in small areas was varied to account for the uncertainty related to small numbers.^44^ Both methods show that for areas with similar scores it is not possible to say which is more deprived, but the 10 most deprived areas are clearly distinguishable from the 10 least deprived areas.

Shrinkage estimation attempts to reduce uncertainty by ‘borrowing strength’ from larger or nearby areas. The deprivation score for a small area will be a weighted combination of the score for the small area itself and the mean of a larger or nearby areas.^3^ The benefit of the shrinkage estimation is that uncertainty would have been minimised and the result is still a single measure. This technique is used for the indices of multiple deprivation across the UK countries.^13 16^ Though, it should still be kept in mind that for areas with similar scores, it is not possible to say with certainty which is more deprived.

Categorical measures of deprivation also reduce uncertainty by splitting the small areas into approximately 4–10 groups based on the continuous deprivation measure. This method ensures that small variations in deprivation have generally no impact on the assignment to a category. It is also straight forward to produce similar categorical measures for both the upper and lower confidence intervals and then compare these to the one based on the actual measure using cross tabulations.^44^ If most areas fall on the diagonal, uncertainty about the deprivation category is small. Any areas that fall off the diagonal would immediately be flagged and researchers can take a closer look at these to determine what drives these results. But even with low levels of uncertainty in the categorical measure, researchers and policy makers should keep in mind that areas with values near to the cut-off points between categories could easily have fallen to either category. As such, belonging into any specific deprivation category may not necessarily be the single correct basis for a policy intervention.

Few research articles explicitly discuss uncertainty in measurement or provide any confidence intervals.^13 16 20 44^ Most often, a categorical deprivation measure is used, but no other methods are applied. Since there are many methods available for dealing with uncertainty, researchers should devote more attention to this issue. Understanding and recognising the uncertainty in measurement provides others better guidance on when and how to use the measure.

## Conclusions

The aim of this paper was to provide clarity and a framework for planning and following through with the process of developing a small-area measure. Our goal is not to define deprivation or to argue that any single methods of constructing an index is universally better. Rather, we emphasise the role of conscious and well-reasoned decision making in the process, the result of which is a measure with clearly defined strengths and limitations.

The construction of small-area composite measures involves a number of stages and decisions, from selecting appropriate data sources, indicators and combining these into a measure, to validating the resultant index and providing uncertainty estimates. For some of these, such as choosing the data source or geographic area level, the options might be quite limited, for others, for example, combining and weighting the indicators, the options are more abundant.

Given the breadth of different options across the stages, there are surprisingly few examples of comparison of deprivation measures that use different methods,^9 11 40 44^ and some of this research compares methods that are only slightly different.^29^ Small variations in the methodology tend to produce little substantive difference on the measured health inequalities.^29^ More substantial differences in measures can also give very similar results in the general population,^9^ but outcomes are more likely to vary for specific subpopulations.^15 36 41^ There is good evidence that different socio-economic indicators are not equally effective in uncovering health inequalities for different population groups^45^ and for this reason, validating indices across populations is crucial, but tends to be neglected in the literature. Overall, we actually have very little knowledge if or what difference the choice between methods (such as equal weighting, expert opinions or empirically driven weights) have on the usefulness of a deprivation measure. Currently, it seems that choices are rooted more in tradition or convention of a research group, rather than any real evidence.

We encourage researchers to consider and compare methods more closely, noting their justification for taking particular decisions and the implications that these may have for the use of the measures. Particularly, more emphasis should be put on validating measures for different population subgroups. A clear and robust decision-making process will result in a measure that is more likely to be used also by other researchers and policy makers.

**What is already known on this subject**

- Small-area composite indices, such as deprivation measures, are a convenient and a popular method of capturing complex or multidimensional concepts, at the whole population level.
- A wide variety of data sources and methods have been used to create small-area deprivation measures.
- Researchers do not often discuss or justify their decision with respect to the data or methods used to develop a small-area measure.

**What this study adds**

- The creation of small-area measures covers multiple stages such as choosing a data source, combining variables into a single measure, validation and uncertainty estimation.
- Researchers should consider and compare the options available at each stage more closely, justifying any decisions and noting the implications these may have for the use of the measures.
- Validation of small-area deprivation measures, especially for different population subgroups, is frequently neglected, but vital for developing a robust measure.
